# Broad-spectrum antitumor analysis of the telomerase activity inhibitor TPCH derived from the human constitutively expressed protein LPTS/PinX1

**DOI:** 10.3389/fonc.2025.1643155

**Published:** 2025-08-15

**Authors:** Hongchang Zhou, Xiaoying Zhang, Yao Wang, Yongqiang Wu, Ling Wang, Chen Hu, Ting Zhang, Hui Zhang, Dian You, Mengli Zhao, Mujun Zhao, Anqi Li, Guangming Chen

**Affiliations:** ^1^ Huzhou Key Laboratory of Precise Prevention and Control of Major Chronic Diseases, School of Medicine, Huzhou University, Huzhou, China; ^2^ Department of Hepatobiliary and Pancreatic Surgery, First Affiliated Hospital of Huzhou University, Huzhou, China; ^3^ Botuvac Biotechnology Co., Ltd, Beijing, China; ^4^ The Institute of Biochemistry and Cell Biology, Chinese Academy of Sciences, Shanghai, China

**Keywords:** LPTS, TPCH, telomerase, telomerase inhibitor, cancer

## Abstract

**Background:**

The human liver-related putative tumor suppressor LPTS/PinX1 is a gene encoding a telomerase inhibitory protein. Overexpression of LPTS/PinX1 protein can inhibit the growth of multiple telomerase-positive cancer cell lines. LPTS/PinX1 has therapeutic potential for cancer.

**Methods:**

We statistically analyzed the level of LPTS/PinX1 protein in 9 cancer cell lines. LPTS/PinX1158-328 (exon 7 of LPTS) was fused with TAT to generate the recombinant protein TPCH. The effects of the TPCH protein on cell growth, senescence and apoptosis in 14 cell lines were analyzed *in vitro* and *in vivo*.

**Results:**

The purified TPCH protein was delivered into cells and inhibited telomerase activity. Also it inhibited the growth of 11 telomerase-positive cancer cell lines, was ineffective in 3 telomerase-negative cell lines *in vitro* and inhibited the growth of MCF-7, A549 and SW480 cell line-derived xenograft (CDX) and liver cancer patient-derived xenograft (PDX) mouse models *in vivo.* The inhibitory effect on the cancer cell growth was negatively correlated with the telomere length. The TPCH protein induced senescence and apoptosis in telomerase-positive cancer cells through the p21 signaling pathway and inhibited the migration of telomerase-positive cancer cells.

**Conclusions:**

The TPCH protein strongly inhibited telomerase activity and suppressed the growth of all tested human telomerase-positive cancer cell lines *in vitro* and *in vivo*. Therefore, it could be developed as a broad-spectrum anticancer agent with low toxicity.

## 1 Introduction

Human telomerase can synthesize telomere repeat sequences by reverse transcription, compensate for telomere loss caused by terminal replication problems, and maintain the stability of chromosomes (1, 2). Telomerase is activated in more than 85% of cancers (3, 4). The activation of telomerase is a key step in cancer development, as it allows cancer cells to avoid senescence and apoptosis (5, 6). Inhibiting telomerase activity can not only induce cancer cell growth inhibition but also eliminate the insensitivity of cancer cells to chemotherapy, cycle arrest and apoptosis (7, 8). Therefore, the development of telomerase inhibitors with high specificity, low toxicity and clear molecular mechanism will have a great impact on anticancer drug research.

LPTS (liver-related putative tumor suppressor) is the only human protein reported to date that can bind to telomerase, inhibit the ability of telomerase to prolong telomeres, and inhibit tumor cell growth *in vitro* and *in vivo (* 9). In contrast to telomerase, LPTS is expressed in normal human tissues but is not expressed or is expressed at low levels in multiple cancer cell lines, such as lung, gastric, colorectal, cervical, breast, and liver (9–14). Increasing the expression of LPTS in cancer cells can effectively inhibit their proliferation and tumorigenicity (15–17). More than 94% of LPTS heterozygous deletion mice developed tumors (18). Therefore, LPTS may be a tumor suppressor gene.

LPTS has 7 exons and 3 transcripts. The longest transcript (LPTS-L) consists of seven exons, and it is identical to PinX1 (PIN2 interacting telomerase inhibitor 1), which encodes a protein consisting of 328 aa that can inhibit the activity of telomerase (19, 20). In a previous study, we found that LPTS/PinX1133–328 aa (exons 6 and 7) were able to inhibit telomerase activity *in vivo* and *in vitro* and induce cellular senescence and apoptosis (21, 22). Thus, the LPTS/PinX1 protein or its active region may have anticancer effects and is expected to be useful for the treatment of cancer.

In the present study, we found that fragment 158–328 aa of LPTS/PinX1 (PC) could strongly inhibit telomerase activity, and its encoding sequence was derived from exon 7 of LPTS/PinX1. Therefore, we constructed the fusion protein TAT-LPTS158-328 (TPCH) of the cell-penetrating peptide TAT (11 amino acid peptide of HIV transcription activator) and PC and tested its inhibitory effect on the growth of 11 telomerase-positive cancer cell lines and 3 telomerase-negative cell lines *in vitro*. The results showed that it could specifically inhibit the growth of multiple telomerase-positive cancer cell lines and that the inhibition was negatively correlated with the telomere length. The TPCH protein induced telomerase-positive cancer cell senescence and apoptosis through the p21 signaling pathway and inhibited telomerase-positive cancer cell migration. In addition, it significantly inhibited the growth of MCF-7, A549 and SW480 CDX tumors and liver PDX tumors *in vivo*. This study is expected to provide a new direction for the development of novel broad-spectrum targeted anticancer drugs.

## 2 Materials and methods

### 2.1 Plasmids, cell lines and reagents

To generate truncated proteins of human LPTS/PinX1, cDNAs were subcloned and inserted into pGEX-6P-1 vectors (Addgene, Watertown, MA, USA) with an N-terminal GST tag or into pTAT vectors (a kind gift from Dr. Steven Dowdy) with an N-terminal TAT tag and a C-terminal 6×His tag. All cells were purchased from the Cell Bank of the National Collection of Authenticated Cell Cultures (Shanghai, China) and cultured in a 37°C humidified incubator containing 5% CO_2_. All cells were grown in DMEM, RPMI-1640 or MoCoy’s 5a medium (Gibco BRL) according to the manufacturer’s instructions. All cells were supplemented with 10% fetal bovine serum (Gibco, Thermo Fisher Scientific Inc., Waltham, MA, USA), 100 units/ml penicillin and 100 μg/ml streptomycin. Tissue samples of patients were obtained from the First People Hospital of Huzhou.

### 2.2 Recombinant protein preparation and purification

The protein was prepared and analyzed according to previous methods (21). Briefly, TAT fusion proteins were purified on Ni^2+^-NTA (nitrilotriacetic acid) agarose columns (Beyotime Biotech. Inc., Shanghai, China), and GST fusion proteins were purified on a glutathione agarose column (Beyotime).

### 2.3 Delivery of fusion proteins into cells

Cells were seeded into 6-well plates, and when the cell density reached 90%, the medium for cultured cells was replaced every two days with fresh medium supplemented with LPTS158-328 (LCH), TPCH or PBS (as a control). The delivery of fusion proteins into the cell nucleus was detected by western blot and immunofluorescence according to previous methods (21). For western blotting, the cytoplasmic and nuclear lysates were prepared with NE-PER Nuclear and Cytoplasmic Extraction Reagents (Thermo Fisher) and then detected using anti-LPTS/PinX1, anti-Lamin1 and anti-GAPDH antibody (Santa Cruz). For immunofluorescence, all treated cells were strained with anti-LPTS/PinX1 and DAPI (4’,6-diamidino-2-phenylindole) and were viewed under a fluorescence microscope (Olympus BX51).

### 2.4 Cell growth, senescence and apoptosis

The cells were treated for 6–10 weeks with PBS, TAT-GFP or TPCH. For cell growth inhibition analysis, the cells were cultured in 96-well plates at a density of 2-3 × 10^3^ cells/well after treatment for 0, 3 or 6 weeks, and cell viability was measured by Cell Counting Kit-8 (CCK8, Dalian Bergolin Biotechnology Co., Ltd, Dalian, China) according to manufacturer’s protocol. Cell apoptosis was analyzed by an Annexin V-FITC kit (Beyotime), and the cells were counted using a FACSCalibur flow cytometer (BD). For cell senescence analysis, the cell and nuclear morphology were viewed under a fluorescence microscope, the senescent cells were detected by a senescence β-Galactosidase Staining Kit (Beyotime) according to the manufacturer’s protocol, viewed under a microscope, and counted by ImageJ software. The senescence- and apoptosis- related proteins were detected using anti-p16 (Sigma-Aldrich), anti-pRB (Santa Cruz), anti-p53 (Santa Cruz), anti-p21 (Proteintech), and anti-C-caspase-3 (Abcam) and anti-βactin (Santa Cruz) antibody.

### 2.5 Telomerase activity and telomere length

The telomeric repeat amplification protocol (TRAP) was used to detect the inhibition of telomerase by various truncated proteins (23). The telomerase-containing fraction was incubated with various LPTS/PinX1 truncated proteins in a particular reaction system (20 mM Tris-HCl (pH 8.3), 1.5 mM MgCl_2_, 63 mM KCl, 0.05% Tween 20, 1 mM EGTA, 50 μM dNTPs, 0.2 μM TS pimer, 0.2 μM ACX pimer, 0.4 mg/mL BSA, 0.04 U/μL Taq DNA polymerase) at 25 °C for 40 min for extension before amplified by PCR. The PCR products were resolved on a 10% polyacrylamide gel and stained by silver staining, and telomerase activity was semiquantified by assaying the band gradient.

To detect telomerase activity in various cells, the same concentration of lysate was added to the reaction system (20 mM Tris-HCl (pH 8.3), 1.5 mM MgCl_2_, 63 mM KCl, 0.05% Tween 20, 1 mM EGTA, 25 μM dNTPs, 0.35 μM TS primer, 0.35 μM ACX primer, 0.4 mg/mL BSA) and incubated at 25 °C for 40 min for extension before amplified and detected by qPCR (TB Green^®^ Premix Ex Taq™ II, Tli RNase H Plus, TaKaRa, Beijing China) and calculation of Ct and ΔCt, as described in reference (21).

The relative telomere lengths (RTLs) of cells or tissues were detected. FFPE DNA kits (Jiangsu Cowin Biotech Co., Ltd., Beijing, China) were used to extract genome from tumor tissue specimens or cell lines before diluting to the same concentration, after which two pairs of primers (GC-Telc and GC-Telg, MRef1F and MRef1R) were added to each sample. The RTL was measured through qPCR (TB Green^®^ Premix Ex Taq™ II, Tli RNase H Plus, TaKaRa) and Ct and ΔCt were calculated as described in reference (21). Genomic DNA was extracted from cells and digested with Hinf I and Rsa I. The DNA was then separated on a 0.7% agarose gel. After the gel was denatured, Southern blotting analysis was performed using α-^32^P-labeled-(TTAGGG)_6_ DNA probe. The hybridization signals were quantified using ImageQuant (Amersham) software, and the average terminal restriction fragment (TRF) length was calculated.

### 2.6 Cell migration assay

For the wound healing assay, the cells (2×10^6^ cells per well) treated with PBS or TPCH were seeded on 6-well plates. 24 h later, the medium was discarded. Then the cells were scratched with a sterile 200 μL pipette tip and washed with PBS and cultured in serum-free medium with 1% bovine serum albumin. The progress of migration was photographed at 0 h, 24 h and 48 h after wounding.

For the transwell migration assay, the cells treated with PBS or TPCH were seeded on 6-well plates. 2×10^6^ cells were added to the top chamber of migration device, and medium containing 20% serum were added to the bottom chamber. After 48 hours, top cells were removed, and bottom cells were washed with PBS, fixed in 4% paraformaldehyde for 30 min, and stained with crystal violet. The number of migrating cells were counted under a microscope.

### 2.7 Animal models and treatment approach

All animal procedures were performed according to protocols approved by the Institutional Guidelines Committee at the Shanghai Jiao Tong University or Huzhou University. For cell line-derived xenograft (CDX) experiments, the breast cancer cell line MCF-7, the lung cancer cell line A549 and the colorectal cancer cell line SW480 were injected (2×10^6^ cells per site) subcutaneously into the right flank of 4- to 5-week-old male BALB/c nude mice (Gem Pharmatech LLC, Nanjing, China). One day after cell implantation, the mice were randomized into different groups (five mice per group) and then injected with PBS, 20 mg/kg TAT-GFP or TPCH via the tail vein. The injections were performed 15 times every 3 days. Tumor size was measured weekly with calipers, and tumor volume was calculated as follows: volume = (length × width^2^) × 0.5. The tumors were then collected and weighed 7 weeks after inoculation.

For the patient-derived xenograft (PDX) experiments, 2 kinds of primary human liver cancer (named hepatocellular carcinoma HCC-1 and HCC-2) xenograft model fragments (2–3 mm in diameter) were inoculated subcutaneously into the right flank of 5- to 6-week-old male BALB/c nude mice. The mice were administered with PBS or 20 mg/kg TPCH via the tail vein 12–14 times, as described above, and the tumor volume was calculated. The tumors were collected, and part of each tissue sample was fixed in paraformaldehyde for further HE staining, TUNEL assay and immunohistochemistry experiments. *In situ* apoptosis was detected by TUNEL assay kits (Thermo Fisher, #C10617) according to the manufacturer’s protocol. For immunohistochemistry assay, Ki67 antibody (Abcam, ab15580) was used as the marker of cell proliferation. Genomic DNA was extracted from parts of these tissues with a Universal Genomic DNA Kit (Cowin, #CW2298), and the RTLs were measured as described above.

### 2.8 Toxicity test

C57BL/6 mice aged 4–5 weeks were selected (GemPharmatech LLC, Nanjing, China). Mice were randomly divided into three groups (9 per group) and then treated with PBS or TPCH (10, 20 or 50 mg/kg) via the tail vein. Injections were performed 20 times every 3 days. The mice were weighed 24 h after the last dose, and the weights were recorded. The mice were euthanized and rapidly dissected, the main organs were isolated and weighed (heart, liver, spleen, lung, kidney, testis), and the organ coefficient (organ weight/body weight ×100%) was calculated. The tissues of heart, liver, spleen, lung, kidney, testis were fixed in 10% formalin solution for histopathological examination.

### 2.9 Statistical analysis

The cell experiments were run in triplicate, and the animal experiments were run in quintuplicate. The n-numbers of the cell experiments were just technical repeats, and these of the animal experiments were independent biological repeats. The data were presented as the means ± SD. Differences between two groups were evaluated using Student’s t test (two-tailed). An ANOVA with a *post-hoc* test was used to compare the three groups and more data. *P* values less than 0.05 were considered statistically significant.

## 3 Results

### 3.1 LPTS/PinX1 gene and protein levels are reduced in most human cancer tissues and cells

**Figure 1:**
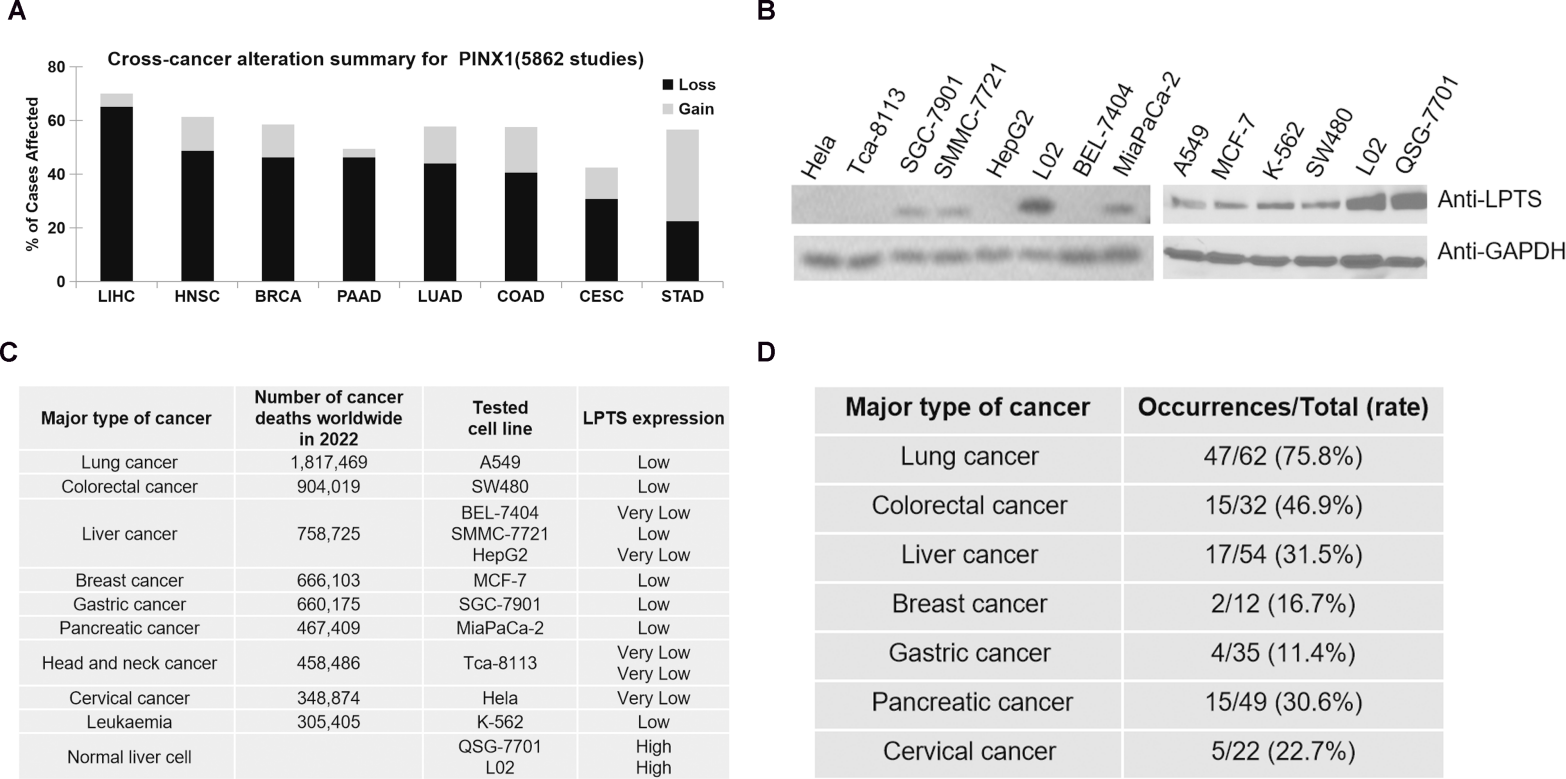
The expression of LPTS/PinX1 decreases in most cancers. **(A)** A pan-cancer view of copy number variation (CNV) distribution in LPTS/PinX1 gene according to data derived form TCGA (The Cancer Genomic Atlas). Y-axis shows the mutation frequency in percentage (including both amplifcation and deletion mutation); x-axis indicates the cancer types. **(B)** The levels of LPTS/PinX1 protein were detected by Western blot., **(C)** Number of cancer deaths worldwide in 2022, by major type of cancer, according to the data derived from the World Global Cancer Observatory of WHO (2022). The levels of LPTS/PinX1 protein were detected in B. **(D)** Tumourigenesis in LPTS/PinX1+/- mice.

The data obtained from the Human Protein Atlas (HPA) suggested that the levels of LPTS/PinX1 mRNA were high in normal tissues (https://www.proteinatlas.org/). However, the frequency of the *LPTS/PinX1* loss often occurred in most cancer tissues, including the colorectum (COAD), head and neck (HNSC), cervical (CESC), liver (LIHC), lung (LUAD), breast (BRCA), pancreas (PAAD) and gastric (STAD) cancer tissues (data from The Cancer Genomic Atlas, https://portal.gdc.cancer.gov/analysis_page?app=CohortBuilder&tab=general) (**Figure 1A**). Then we examined LPTS/PinX1 protein level. As shown in **Figure 1B**, in all 11 cancer cell lines, the level of LPTS/PinX1 protein decreased compared with that in the normal liver cell lines L02 and QSG-7701. We concluded that in these fatal cancers, the level of LPTS/PinX1 protein was low (data derived from the World Global Cancer Observatory of WHO 2022, https://gco.iarc.fr/today/en/fact-sheets-cancers) (**Figure 1C**). Then, we used the LPTS/PinX1+/- mice to study the incidence of cancer in these mice. We found the LPTS/PinX1+/- mice exhibited increased rates of tumorigenesis. The occurrences of lung, colorectum, liver and pancreas cancer were approximately 75.8%, 46.9%, 31.5% and 30.6%, but stomach cancer rarely occurred (**Figure 1D**). Together, these results indicated that LPTS/PinX1 level is reduced in most human cancer tissues and cells and that LPTS/PinX1 is a major tumor suppressor.

### 3.2 LPTS/PinX1158–328 strongly inhibited telomerase activity and cell proliferation

**Figure 2:**
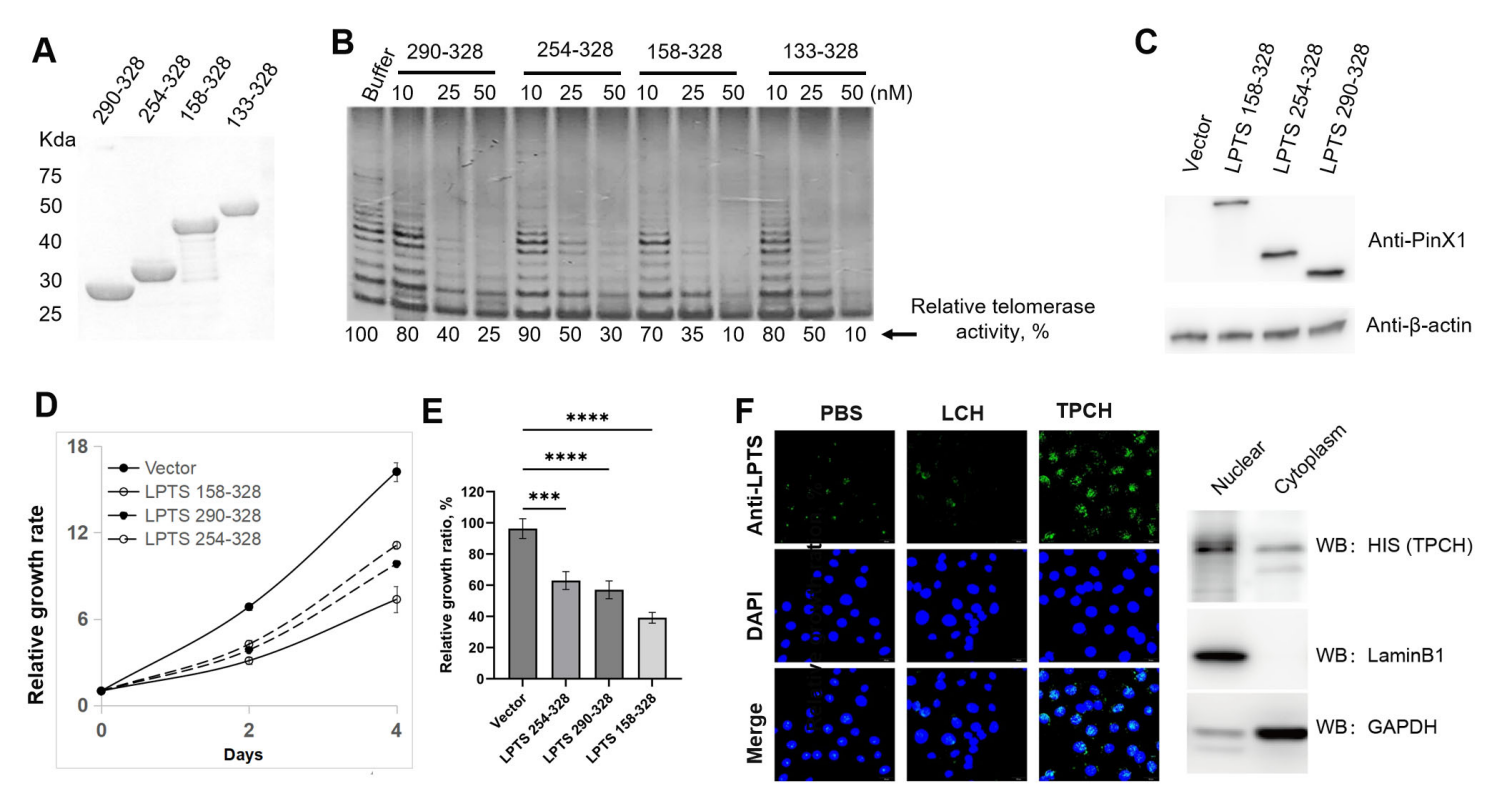
LPTS/PinX1158–328 strongly inhibited telomerase activity and cell proliferation. **(A)** SDS-PAGE analysis of purified C-terminal LPTS/PinX1 proteins with GST tag. **(B)** The inhibition of telomerase activity by different C-terminal LPTS/PinX1 proteins *in vitro* was compared via a TRAP assay. The telomerase-containing fraction was prepared from the cell line Hela. **(C)** Western blot analysis of Hela cells overexpressing C-terminal LPTS/PinX1 proteins fused to the N-terminal of the AcGFP1 protein. **(D)** CCK8 assay of cell growth after overexpression of different C-terminal LPTS/PinX1 proteins (n=3). **(E)** Inhibitory effect of different C-terminal LPTS/PinX1 proteins on the growth of Hela cells compared with that of the corresponding vector (n=3). *** represents P < 0.001, **** represents P < 0.0001. **(F)** The purified recombinant protein TPCH entered the Hela cell nucleus, as detected by immunofluorenscence and Western blotting.

LPTS/PinX1 inhibits telomerase activity through the C-terminal region. We compared the inhibitory effects of three reported C-terminal LPTS/PinX1 fragments with the novel discovered fragments LPTS/PinX1158-328 (from exon 7 of LPTS/PinX1) on telomerase activity. We constructed four GST fusion proteins, which were highly expressed in *E. coli* BL21 and purified (**Figure 2A**). The TRAP results indicated that, at the same concentration of the four LPTS/PinX1 proteins, LPTS/PinX1158–328 showed the strongest inhibition of telomerase activity *in vitro*, and the LPTS/PinX1158–328 protein almost completely inhibited telomerase activity at a concentration of 50 nM (**Figure 2B**). We then overexpressed three kinds of LPTS/PinX1 fragments (158-328, 254-328, 290-328) in Hela cells (**Figure 2C**). Then, their growth rate was examined using the CCK8 assay, and the curves showed that the growth rates of cells overexpressing the three LPTS/PinX1 fragments (254-328, 290-328, 158-328) were approximately 66.5%, 60% and 41.8%, respectively, of those of the control group (**Figures 2D, E**). The above results indicated that LPTS/PinX1158–328 had a greater ability to inhibit telomerase activity and cell growth than the other LPTS/PinX1 fragments. We fused the LPTS/PinX1158–328 region with TAT to generate the TAT-LPTS158-328 (TPCH) fusion protein. The immunofluorescence and Western blot results indicated that the fusion protein TPCH could enter Hela cells and accumulate mainly in the nucleus. (**Figure 2F**).

### 3.3 TPCH inhibited the growth of cancer cells in a telomere length-dependent manner

**Figure 3:**
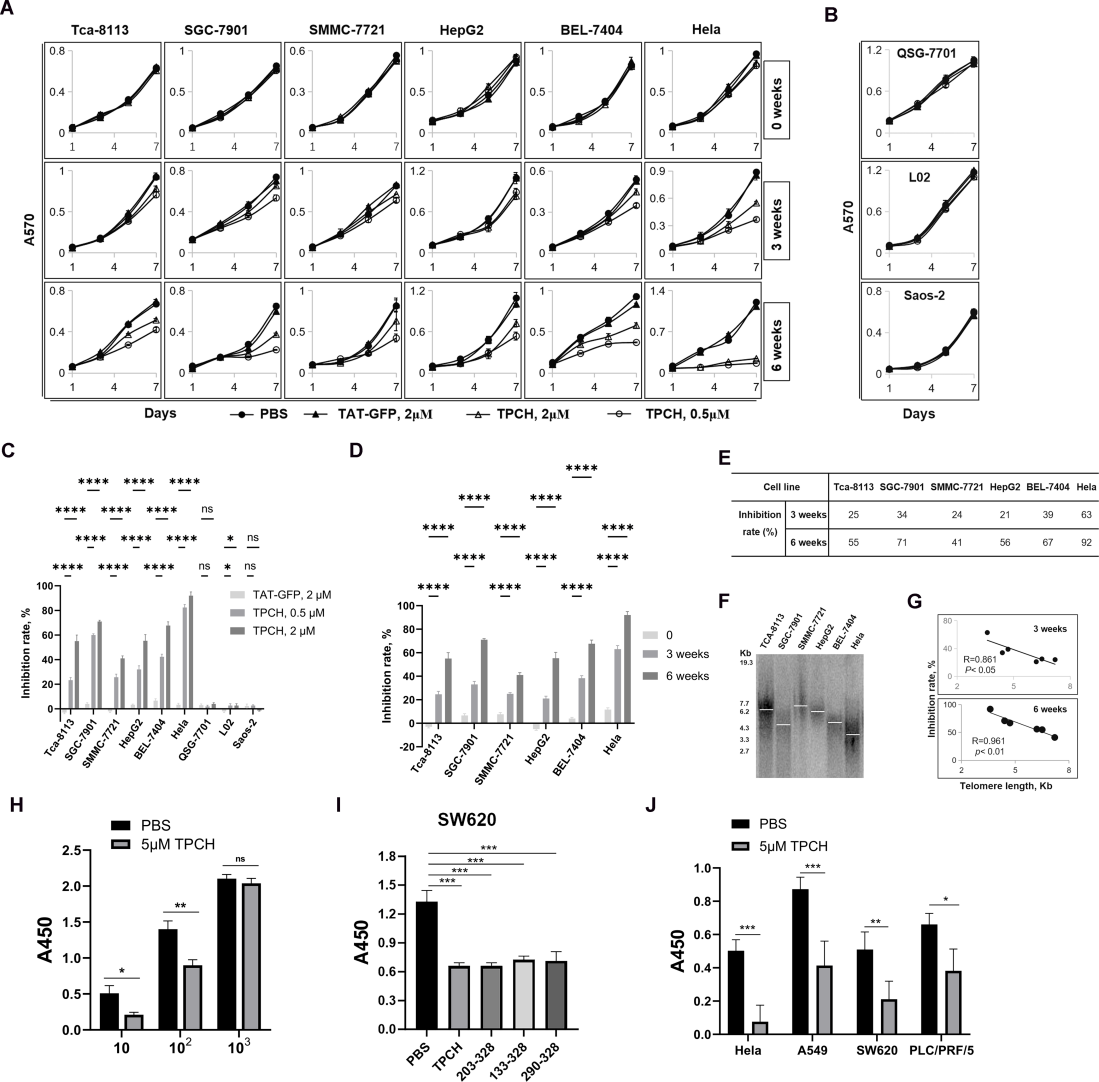
TPCH suppressed the growth of cancer cells in a dose-, time- and telomere length-dependent manner. **(A)** The growth of telomerase-positive cancer cell was measured by MTT assay. The cells were treated with PBS, 2 μM TAT-GFP, 0.5 μM or 2 μM TPCH for the indicated times (n=3). **(B)** The growth of telomerase-negative cell was measured by MTT assay. The cells were treated with PBS, 2 μM TAT-GFP, 0.5 μM or 2 μM TPCH for the indicated times (n=3). **(C)** Histograms showing the growth inhibition rate of cells treated with 2 μM TAT-GFP, 0.5 μM TPCH or 2 μM TPCH for 6 weeks compared with that of the PBS group. **(D)** Histograms showing the growth inhibition rate of cells treated with 2 μM TPCH protein for 0, 3 or 6 weeks compared with that of the PBS group. **(E)** Growth inhibition rate of cells treated with 2 μM TPCH protein for 3 or 6 weeks compared with that of the PBS group. **(F)** Southern blot analysis of the telomere length of cancer cells before treatment with the TPCH protein. a-^32^P-labelled TTAGGGA repeats were used as probes. **(G)** Pearson’s correlation coefficient calculation between telomere length measured by Southern blot and inhibition rate. **(H)** CCK8 assay of SW620 cell was detected in different cell culture numbers (10, 10^2^, 10^3^, respectively). The cells were treated with PBS or 5 μM TPCH for 14 days (n=3). **(I)** CCK8 assay of SW620 cell. The cells were treated with PBS or 5 μM different C-terminal LPTS/PinX1 proteins (TPCH, 203-328, 133-328, 290-328, respectively, n=3) for 14 days. **(J)** CCK8 assay of telomerase-positive cancer cell lines. The cells were treated with PBS or 5 μM TPCH for 14 days (n=3). Here, ns represents not significant, * represents P < 0.05, ** represents P < 0.01, *** represents P < 0.001, **** represents P < 0.0001.

To detect the inhibitory effect of TPCH protein on cell growth we selected 6 telomerase-positive cancer cell lines and 3 telomerase-negative cell lines. Compared with PBS and TAT-GFP, 0.5 μM or 2 μM TPCH inhibited the growth of all telomerase-positive cancer cell lines after 3 weeks of treatment and the inhibition was significantly enhanced after 6 weeks of treatment (**Figure 3A**). The TPCH protein had no significant effect on telomerase-negative cells, including the telomase-negative cancer cell line Saos-2 and normal liver cell lines L02 and QSG-7701 (**Figure 3B**). After 6 weeks of treatment, the growth inhibition rate of 6 telomerase-positive cancer cell lines was significantly higher when using 2 μM TPCH protein compared to 0.5 μM TPCH protein, indicating that a high concentration of TPCH protein can more effectively inhibit the growth of cancer cells (**Figure 3C**). Similarly, longer TPCH treatment time was associated with a greater inhibitory effect on cancer cells (**Figure 3D**). After 3 weeks of treatment, the growth inhibition rate of the 2 μM TPCH-treated group of Hela cells reached 63%, and after 6 weeks of treatment, the inhibition rate reached 92%, almost completely inhibiting the growth of Hela cells (**Figure 3E**). These data suggested that the TPCH protein inhibited the growth of telomerase-positive cancer cells in a dose- and time-dependent manner.

The TPCH protein inhibited the growth of 6 telomerase-positive cancer cell lines, but the inhibitory effect was variable (**Figure 3E**). We measured the telomere length of 6 telomerase-positive cancer cells by TRF Southern blot. The results showed that initial telomere length differed among these 6 cell lines, and the inhibition effect of the TPCH protein on cell growth was negatively correlated with initial telomere length (**Figures 3F, G**). Therefore, we suggest that the inhibition of TPCH may be more effective on the growth of telomerase-positive cancer cells with shorter initial telomere length.

We also detected the growth inhibitory effect of TPCH in cancer cells after a short period of treatment through the CCK8 assay. SW620 cells were plated at varying densities (10, 10^2^, and 10^3^ cells per well) and incubated for 14 days. The lower cell density showed more obvious inhibitory effect on SW620 cell growth (**Figure 3H**). Compared with other types of LPTS/PinX1 fragments (203-328, 133-328, 290-328), TPCH indeed inhibited the growth of SW620 cells more effectively (**Figure 3I**). Then multiple cancer cell lines were plated at 10 cells per well and incubated for 14 days to detect the growth kinetics. Compared with PBS, 5 μM TPCH significantly inhibited the growth of Hela, A549, SW620 and PCL/PRF/5 cell lines. The growth inhibition rate of the 5 μM TPCH-treated group of Hela cells reached 84.7%, almost completely inhibiting cell growth (**Figure 3J**). The growth inhibition rate of A549, SW620 and PCL/PRF/5 cell lines were approximately 52.6%, 58.6% and 42.1%, respectively. All these results suggested that the TPCH protein could inhibit the growth of multiple telomerase-positive cancer cells in a short time and the inhibitory effect was inversely correlated with the cell density.

### 3.4 The TPCH protein induced telomerase-positive cancer cell senescence and apoptosis

**Figure 4:**
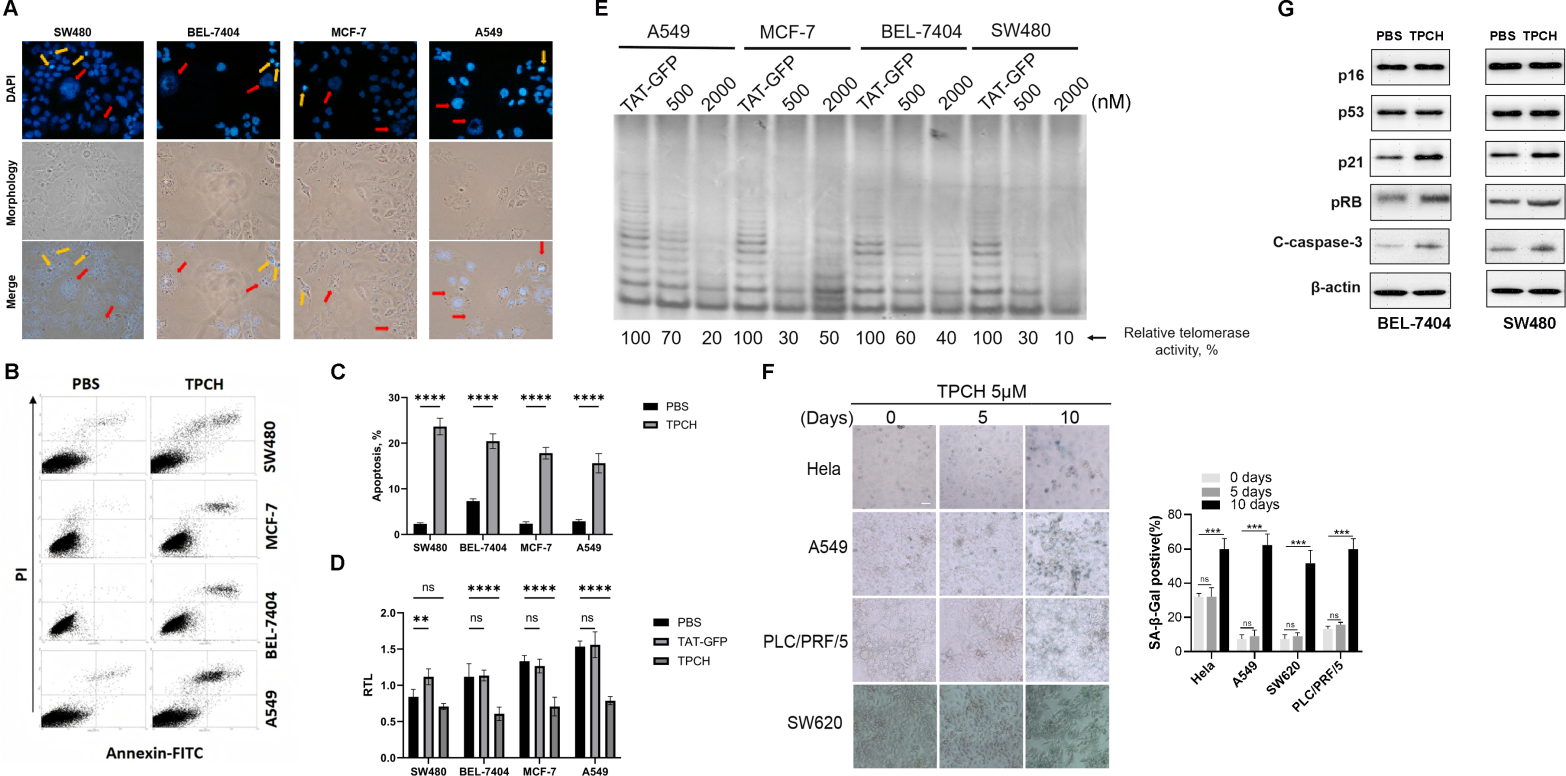
TPCH induced telomerase-positive cancer cell senescence and apoptosis. **(A)** Photographs of the above cells treated with TPCH for 6 weeks. The red arrows indicate the senescent cells, and the yellow arrows show apoptotic cells. **(B)** Apoptosis of the cells described in A was measured by flow cytometry. **(C)** Histograms showing the percentage of apoptosis cells in B. **(D)** Histograms showing the RTLs of SW480, BEL7407, MCF-7 and A549 cell lines treated with TAT-GFP or TPCH for 6 weeks, which were measured by qPCR. **(E)** The telomerase activity of SW480, BEL7407, MCF-7 and A549 cell lines treated with TAT-GFP, 500 nM or 2000 nM TPCH for 6 weeks was compared via a TRAP assay. **(F)** Representative images of cell senescence in Hela, A549, PCL/PRF/5 and SW620 cells treated with PBS or TPCH for indicated times evaluated by staining for β-galactosidase (scale bar, 20 μm). Histograms showing the percentage of senescent cells. **(G)** Western blot analysis of the senescence and apoptosis pathways in BEL7404 and SW480 cells treated with TPCH for 6 weeks. Here, ns represents not significant,** represents P < 0.01, *** represents P < 0.001, **** represents P < 0.0001.

After treatment with the TPCH protein for 6 weeks, some cells of BEL-7404, SW480, MCF-7 and A549 showed an increase in size, failed to form clusters and had enlarged or weakly stained nuclei (**Figure 4A**, red arrow), thus revealing the characteristics of senescent cells. In addition, apoptosis was observed in all four types of cell lines, which became small and round with fragmented nuclei (**Figure 4A**, yellow arrows). Then, we analyzed apoptosis by flow cytometry. Compared with the control group, all four types of cells treated with the TPCH protein exhibited late apoptosis (**Figure 4B**). The apoptosis percentage of SW480, BEL-7404, MCF-7 and A549 cell lines were approximately 23.6%, 20.4%, 17.8% and 15.6%, respectively (**Figure 4C**). In addition, qPCR was used to verify that the telomere length of each cell line was significantly shortened after TPCH treatment (**Figure 4D**). The TRAP results also indicated that, the TPCH protein inhibited telomerase activity of SW480, BEL-7404, MCF-7 and A549 cell lines (**Figure 4E**). These data suggested that the TPCH protein could shorten telomere length and induce cell apoptosis.

We also evaluated the cellular senescence of Hela, A549, PLC/PRF/5 and SW620 cell lines after a short period of TPCH treatment. After 10 days treatment, more than half of TPCH group Hela, A549, PLC/PRF/5 and SW620 cell lines became large, flat, had blurred edges and SA-β-gal-positive staining, which are typical changes related to cellular senescence, and few senescent cells were found in PBS group (**Figures 4F**). The above results indicated that the TPCH protein may induce telomerase-positive cancer cell senescence and apoptosis. Then the Western blot assays were performed to examine the levels of senescence- and apoptosis-related proteins. The results showed that the levels of p21, pRB and Cleaved Caspase-3 (C-caspase-3) increased, while that of p16 and p53 did not clearly change (**Figure 4G**). Thus, these results indicated that the TPCH protein may induce telomerase-positive cancer cell senescence and apoptosis via the p21 pathway.

### 3.5 The TPCH protein inhibited cell migration

**Figure 5:**
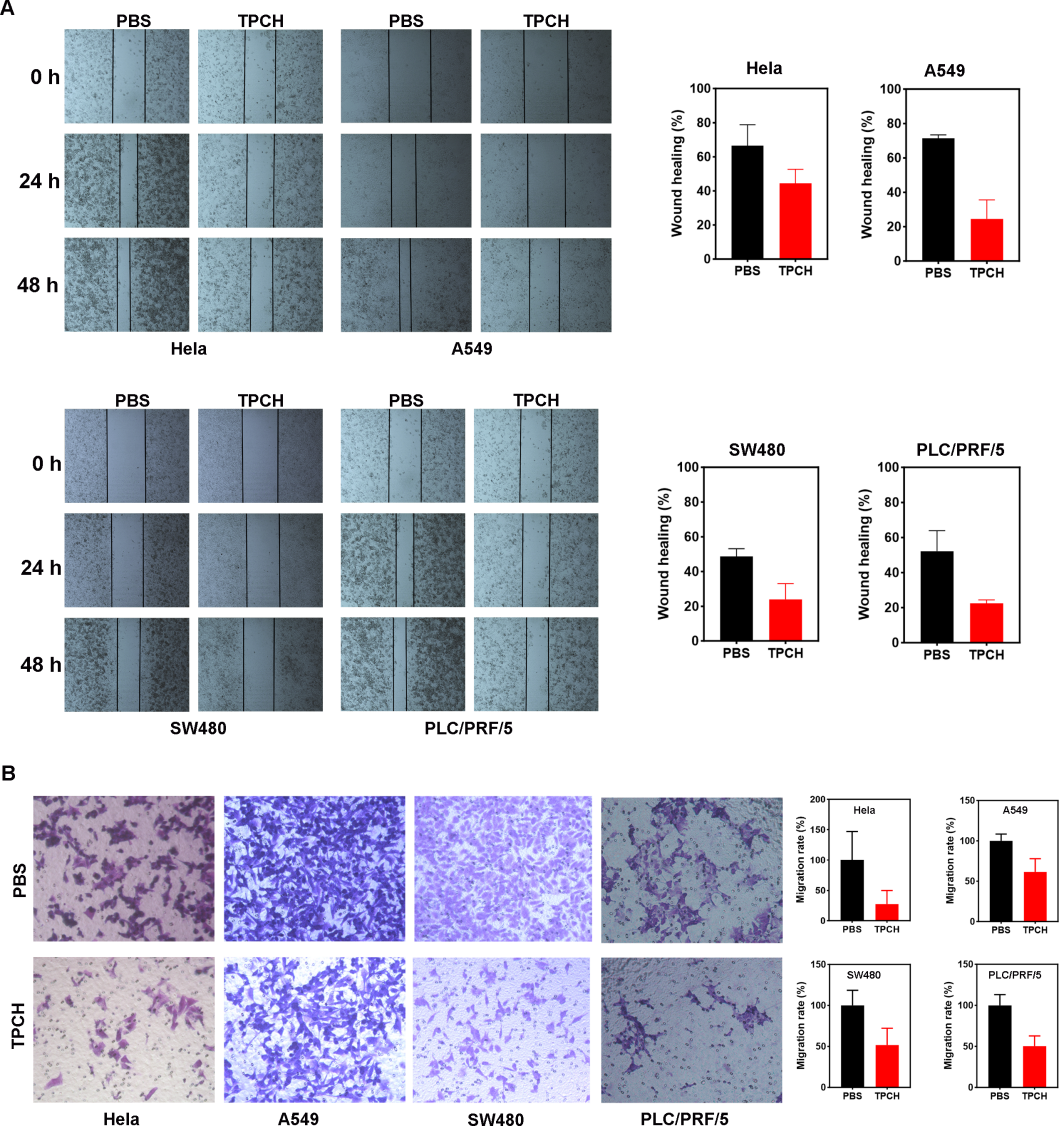
Wound healing and transwell assays to determine cell migration. **(A)** Wound-healing assays were performed at 0, 24 and 48h in Hela, A549, SW480, and PLC/PRF/5 cells in TPCH treated cells and PBS treated cells were used as controls (scale bar, 200 μm). **(B)** Transwell assays were performed in Hela, A549, SW480, and PLC/PRF/5 cells in TPCH treated cells and PBS treated cells were used as controls (scale bar, 100 μm). Results were showed as the mean ± standard deviation (n=3).

Studies have found that in nasopharyngeal carcinoma stem cells (CSCs), PinX1 inhibited epithelial-mesenchymal transformation of nasopharyngeal carcinoma CD133-positive stem cells by regulating the transcriptional inhibition of Snail1, Twist1 and Zeb1 mediated by P53/mir-200b, thereby inhibiting cell proliferation, migration and invasion (24). We then investigated the effects of TPCH protein on the metastatic potential of Hela, A549, SW480 and PCL/PRF/5 cell lines. A wound healing assay showed that the TPCH protein significantly delayed the cell motility to the wound area of Hela, A549, SW480 and PCL/PRF/5 cell lines compared with PBS (**Figure 5A**). After the TPCH protein treatment for 48 hours, the wound healing rate of A459 cells was only 24.5%, while that of the control group reached 71.3%. And the wound healing rates of Hela, SW480 and PCL/PRF/5 cell lines showed similar results. A transwell assay further revealed that the TPCH protein dramatically decreased the migration ability of Hela, A549, SW480 and PCL/PRF/5 cell lines (**Figure 5B**). After treatment with the TPCH protein, Hela cells showed significant cell migration inhibition with only 27.2% of migrated cells compared to control cells. While A549, SW480 and PCL/PRF/5 cell lines treated with the TPCH protein showed cell migration rates of 50–60%. Thus, overall results showed that the TPCH protein inhibited the migration of telomerase-positive cancer cells.

### 3.6 The TPCH protein suppressed tumor growth in xenografted mice

**Figure 6:**
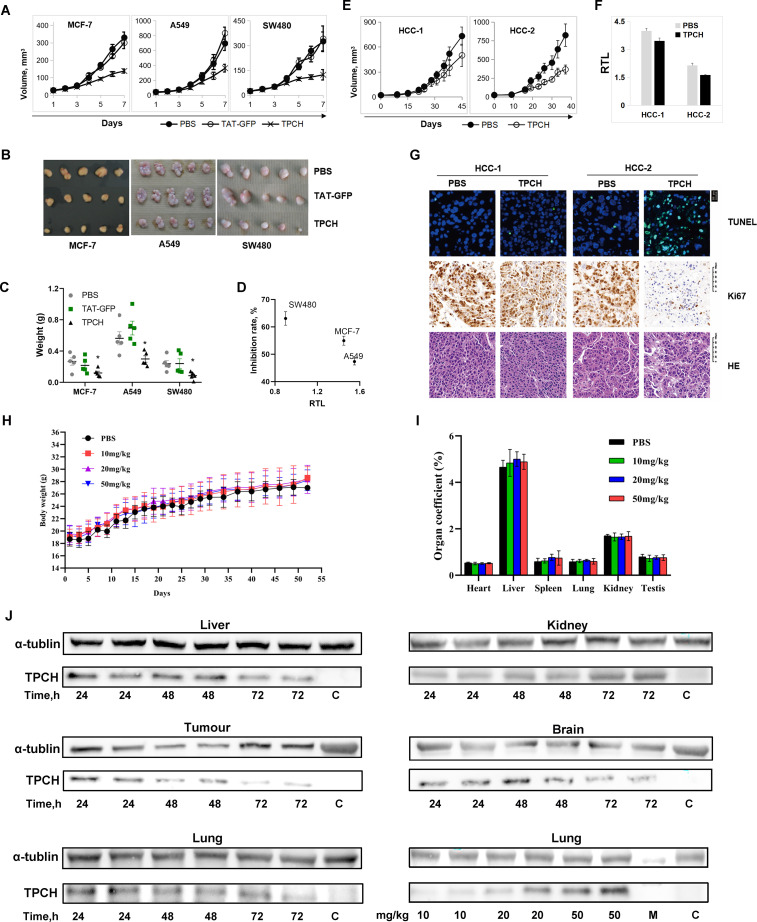
The TPCH protein suppressed the xenograft tumor growth *in vivo.* **(A)** TAT-GFP or TPCH protein was delivered into xenograft mice bearing MC7-7, A549 and SW480 cell tumors, and the tumor volumes were calculated weekly (n=5). **(B)** Photographs of the tumors after xenografting for 7 weeks. **(C)** The average tumor weights were determined. * represents P < 0.05. **(D)** The relationship between RTL measured before TPCH treatment and the inhibition rate. **(E)** TPCH protein was delivered into liver cancer PDX mice, and the tumor volumes were calculated (n=5). **(F)** Histograms showing the RTLs of HCC-1 and HCC-2 PDX cells, which were measured by qPCR. **(G)** Photographs of tumor tissues from HCC-1 and HCC-2 PDXs stained with TUNEL, Ki67 and HE. **(H)** The changes in body weight after the TPCH protein treatment (n=5). **(I)** Organ coefficient of mice. The heart, liver, spleen, lung, kidney and testis were collected and weighed (n=5). **(J)** Western blotting analysis of the TPCH proteins reached the tumors and organs.

We then examined the inhibitory effect of the TPCH protein on tumor growth *in vivo* through CDX and PDX models. In the A549, SW480 and MCF-7 CDX models, a significant cessation of tumor growth was observed in the TPCH protein group compared to the PBS group, and the weight inhibition rates were 53.1%, 36% and 34.5%, respectively, but TAT-GFP groups had no obvious effect on tumor growth compared to the PBS group (**Figures 6A-C**). Moreover, the inhibition effect of the TPCH protein on tumor growth was also negatively correlated with initial telomere length (**Figure 6D**). In the PDX models, after administration with TPCH both HCC-1 and HCC-2 tumors grew slowly, the inhibition rates were 36.6% and 51.2%, respectively, and their RTLs were decreased (**Figures 6E, F**).

Subsequently, we observed four HE-stained images, all of which showed large, deeply stained nuclei, confirming that the PDX remained a tumor cell during the experiment (**Figure 6G**). The Ki67 immunohistochemistry results indicated that HCC-2 tumors after administration with TPCH showed a significant reduction of staining, consistent with the growth curve results which suggested that the cell proliferation signal was significantly inhibited by the TPCH protein (**Figure 6G**). In contrast, due to limited telomere shortening, the proliferation signal in HCC-1 with the treatment of TPCH remained at the same level as the PBS group (**Figure 6G**). Additionally, TUNEL analysis revealed a significant amount of green fluorescence in HCC-2 with the treatment of TPCH, indicating cell apoptosis. In both PBS groups and HCC-1 with TPCH group, only sporadic green fluorescence was observed (**Figure 6G**). The result of HCC-1 with the treatment of TPCH indicated that the classic phenotype of telomere shortening to the limit (slowed proliferation, apoptosis), suggesting that TPCH’s therapeutic effect on the PDX is achieved by inhibiting telomerase activity and shortening telomere length (**Figures 6F, G**). These results indicated that the TPCH protein could inhibit the growth and induce apoptosis of HCC PDX.

### 3.7 *In vivo* toxicity evaluation of the TPCH protein

We also evaluated the long-term toxicity of the TPCH protein on C57BL/6 mice. After 60 days of treatment, there was no significant difference in mouse weights and organ coefficients of heart, liver, spleen, lung, kidney and testis between the TPCH groups and the PBS group (**Figure 6H, I**). The tissues of heart, liver, spleen, lung, kidney and testis had no pathological change in TPCH group (data not shown). These data suggest that the 50mg/kg TPCH protein had no significant toxicity on mice within 60 days administration and the 50mg/kg TPCH were higher than the used 20mg/kg for the mouse experiments above. Western blotting results clearly showed that the TPCH protein actually reached the tumors and was retained in tumors for 72 hours (**Figure 6J**). However, the TPCH protein delivery was not tumor-specific, there were protein signals in liver, lung, kidney and brain (**Figure 6J**).

## 4 Discussion

Activated telomerase is present in most types of cancer cells, therefore, telomerase inhibitors are considered as a broad-spectrum anti-tumor agent. GRN163L (Imetelstat), a synthetic small molecular telomerase inhibitor, has been reported to inhibit the growth of multiple cancer cell lines *in vitro*, such as myeloma, hepatoma, breast cancer, lung cancer and pancreatic cancer (25–29), while *in vivo* there are massive side effects due to telomerase activity in adult stem cells and lymphocytes. LPTS/PinX1, a human liver putative tumor suppressor, could inhibit the cancer cell growth by binding to the hTERT/hTR complex directly (9, 19). Overexpression of LPTS/PinX1 could inhibit the tumor cell growth in the colorectum, breast, liver, stomach and lung, etc. (15, 16, 20, 30, 31). Our previous study found that the LPTS/PinX1133–328 could inhibit the growth of 7 hepatocellular carcinoma cell lines and 1 hepatic adenocarcinoma cell line (22), emphasizing its potential as a broad-spectrum drug candidate for liver cancer treatment. In this study, we demonstrated that TPCH, which derived from LPTS/PinX1, could inhibit the growth of 11 telomerase-positive tumor cells belonging to 9 kinds of immortal human cancer cell lines *in vitro*, and 3 types of CDX and 2 types of PDX *in vivo*. Moreover, after delivery, the TPCH protein could be detected in liver, lung, kidney, brain and tumors over 72 hours (**Figure 6J**). These data indicate that TPCH may be a broad spectrum for cancer treatment.

TPCH inhibited the growth of all tested telomerase-positive cancer cells, but was ineffective in 3 telomerase-negative cell lines, suggesting that the anti-cancer ability of TPCH is highly specific by targeting telomerase. Telomerase inhibitors could reactivate telomere shortening and cause replicative senescence and apoptotic cell death of tumor cells (32). SW480, BEL-7404, MCF-7 and A549 cancer cell lines treated with TPCH showed typically senescent and apoptotic morphology (**Figure 4A**). After long-term treatment with TPCH, the RTLs of SW480, BEL-7404, MCF-7 and A549 got shorter, which activated senescence and apoptosis signaling, such as p21, pRB and C-caspase-3 (**Figures 4D, G**). The results indicate that TPCH probably induces cancer cell senescence and apoptosis by targeting telomerase.

Telomerase inhibitors can be divided into 4 categories according to their action (33, 34): (1) nucleoside analogs such as 6-thio-2’-deoxyguanosine (6-thio-dG) (35); (2) TERC targeting inhibitors such as GRN163L (Imetelstat) (36); (3) G-quadruplex inhibitors such as Telomestatin (37); (4) Non-nucleoside small molecule inhibitors such as BIBRI532 (38). Imetelstat is the only telomerase-specific anticancer drug that has progressed to clinical trials (36). However, most of the clinical trial of Imetelstat on different types of cancers have failed, which may be due to their toxicity and side effects (39). In March, 2024, the U.S. Food and Drug Administration (FDA) Oncologic Drugs Advisory Committee voted in favor of the clinical benefit/risk profile of imetelstat for the treatment of transfusion-dependent anemia in adult patients with low-to-intermediate-1 risk myelodysplastic syndromes (40). The development of telomerase inhibitors with high specificity and low toxicity is demanding. TPCH, a peptide derived from the human protein LPTS/PinX1, is possibly an ideal telomerase protein inhibitor that can interact strongly with telomerase while have low toxicity. 50 mg/kg TPCH injection every 3 days did not affect the physiological status of mice within 60 days of administration in this chronic toxicity test (**Figures 6H, I**). Moreover, in our previous acute toxicity test, the median lethal dose of TAT-LPTS-LC was 174 mg/kg, significantly higher than the 5 mg/kg used in all BEL-7404 cell xenografted mice (21). All these data sufficiently indicated that the TPCH protein might be safe for anti-cancer treatment. And our cell and animal studies also showed that increasing the dose of the TPCH protein might more effectively inhibit the growth of cancer cells.

Although TPCH inhibited cancer cell growth with high specificity and low toxicity, the time for the TPCH to exert the anti-tumor effect is extended, and its inhibition rate of tumor volumes in CDX/PDX models was only about 50%. The efficacy of TPCH on tumor treatment may be improved by 3 methods: (1) Inhibition of telomerase activity might be enhanced by the application of telomerase inhibitors combined with other anti-cancer drugs such as doxorubicin and paclitaxel (41, 42). (2) The anti-tumor effect of telomerase inhibitors largely depends on the initial telomere length of the tumor tissue, such as the telomerase inhibitor imetelstat (43). In the cell lines with longer initial telomere length, the growth inhibition of TPCH was limited (**Figures 3F, G**). Therefore, TPCH might be more effective to cancer cells with short telomeres, which could provide flexible choices for clinical application of TPCH and personalized treatment of cancer patients. (3) There is a delayed period between the initial treatment of telomerase inhibition and the emergence of efficacy, which makes it impossible for telomerase inhibitors to produce rapid antitumor effects. The inhibitory effect of the TPCH protein on cancer cell growth was negatively correlated with the cell density (**Figure 3H**). Therefore, malignancy patients with recurrent or metastatic lesions after treatment might benefit from TPCH due to the small lesions and a longer treatment period.

In conclusion, we constructed a recombinant protein TAT-LPTS158-328 (TPCH). The TPCH protein could enter cells, inhibit multiple telomerase-positive cancer cells’ growth and migration, and induce cell senescence and apoptosis. In addition, the TPCH protein is ineffective against telomerase-negative cells and has extremely low toxicity to experimental animals. Our study suggested that the TPCH protein has the potential for further investigations as a broad-spectrum anticancer drug.

## Data Availability

The original contributions presented in the study are included in the article/supplementary material. Further inquiries can be directed to the corresponding author.
